# Oncological outcomes in fertility-sparing treatment in stage IA-G2 endometrial cancer

**DOI:** 10.3389/fonc.2022.965029

**Published:** 2022-09-16

**Authors:** Carlo Ronsini, Lavinia Mosca, Irene Iavarone, Roberta Nicoletti, Davide Vinci, Raffaela Maria Carotenuto, Francesca Pasanisi, Maria Cristina Solazzo, Pasquale De Franciscis, Marco Torella, Marco La Verde, Nicola Colacurci, Luigi Cobellis, Giuseppe Vizzielli, Stefano Restaino

**Affiliations:** ^1^ Department of Woman, Child and General and Specialized Surgery, Obstetrics and Gynecology Unit, University of Campania “Luigi Vanvitelli”, Naples, Italy; ^2^ Department of Obstetrics, Gynecology, and Pediatrics, Obstetrics and Gynecology Unit, Udine University Hospital, Udine, Italy; ^3^ Dipartimento di Area Medica (DAME), Udine University Hospital, Udine, Italy

**Keywords:** endometrial cancer, fertility, pregnancy outcomes, medroxyprogesterone acetate, levonorgestrel intrauterine device (IUD)

## Abstract

**Background:**

The gold standard treatment for early-stage endometrial cancer (EC) is hysterectomy with bilateral salpingo-oophorectomy (BSO) with lymphadenectomy. In selected patients desiring pregnancy, fertility-sparing treatment (FST) can be adopted. Our review aims to collect the most incisive studies about the possibility of conservative management for patients with grade 2, stage IA EC. Different approaches can be considered beyond demolition surgery, such as local treatment with levonorgestrel-releasing intra-uterine device (LNG-IUD) plus systemic therapy with progestins.

**Study design:**

Our systematic review was performed according to the Preferred Reporting Items for Systematic Reviews and Meta-Analyses (PRISMA) statement. PubMed, EMBASE, and Scopus databases were consulted, and five studies were chosen based on the following criteria: patients with a histological diagnosis of EC stage IA G2 in reproductive age desiring pregnancy and at least one oncological outcome evaluated. Search imputes were “endometrial cancer” AND “fertility sparing” AND “oncologic outcomes” AND “G2 or stage IA”.

**Results:**

A total of 103 patients were included and treated with a combination of LNG-IUD plus megestrol acetate (MA) or medroxyprogesterone acetate (MPA), gonadotrophin-releasing hormone (GnRH) plus MPA/MA, hysteroscopic resectoscope (HR), and dilation and curettage (D&C). There is evidence of 70% to 85% complete response after second-round therapy prolongation to 12 months.

**Conclusions:**

Conservative measures must be considered temporary to allow pregnancy and subsequently perform specific counseling to adopt surgery. Fertility-sparing management is not the current standard of care for young women with EC. It can be employed for patients with early-stage diseases motivated to maintain reproductive function. Indeed, the results are encouraging, but the sample size must be increased.

## Introduction

Endometrial cancer (EC) is the most common gynecological malignancy, with 319,500 cases each year and over 76,000 deaths annually. EC represents the most frequent tumor affecting the uterus, and it may depend on the administration of unopposed estrogens (1). Moreover, in Western countries, the age of the first pregnancy has shown an opposite trend, raising its threshold. The gold standard technique for the detection and determination of both cervical invasion and myometrial infiltration is transvaginal ultrasound (TVS) (2). The gold standard treatment is hysterectomy with bilateral salpingo-oophorectomy (BSO) with lymphadenectomy (3, 4).

Nevertheless, in selected cases of patients desiring pregnancy, fertility-sparing treatment (FST) can be proposed. Nowadays, inclusion criteria are rigorous and concerned: women younger than 40 years who plan to conceive as soon as possible after remission, histology of grade 1 EC, endometrioid histotype with positive hormone receptor (type I), tumor diameter <2.0 cm, International Federation of Gynecology and Obstetrics (FIGO) stage IA with neither myometrial nor adnexal involvement, negative lymphovascular space invasion (LVSI), diffuse immunohistochemical expression of progesterone receptors on endometrial biopsy, and stage of disease verified by magnetic resonance imaging (MRI) (4). This kind of patient shows excellent 5-year progression-free survival (PFS) rates—95%—if the tumor is grade 1 with overall survival (OS) rate of 90% (5). In contrast, treating patients with grade 2 (G2) is much more controversial. Beyond the current guidelines, many referral centers propose thrombospondin (TSP) for this type of patient (6). Our review aims to evaluate the oncologic outcomes of patients affected by IA G2 EC who have been administered with FST.

## Methods

**Figure 1:**
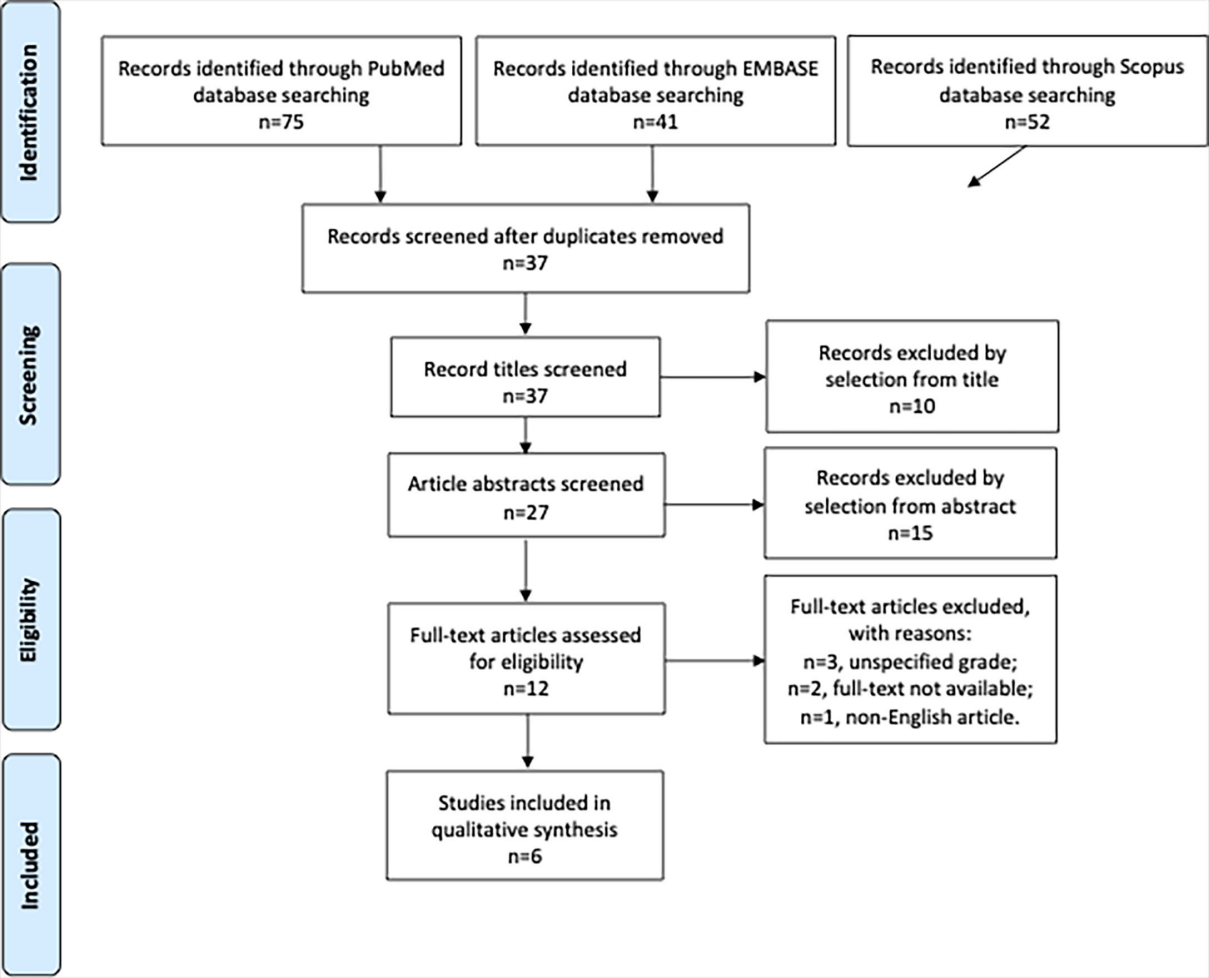
PRISMA 2009 flow diagram.

This systematic review was performed according to the Preferred Reporting Items for Systematic Reviews and Meta-Analyses (PRISMA) statement (7). We systematically searched articles about oncological outcomes in FST of EC FIGO stage IA G2 in PubMed, EMBASE, and Scopus databases in April 2022 from the first publication. We made no restrictions on the country. We considered only entirely English-published studies. Search imputes were “endometrial cancer” AND “fertility-sparing” AND “oncologic outcomes” AND “G2 or stage IA”. Study selection was made independently by RN and DV. In case of discrepancy, LM decided on inclusion or exclusion. Inclusion criteria were studies including patients with EC stage IA G2; studies reporting at least one oncological outcome of interest—OS, disease-free survival (DFS), recurrence rate (RR), and complete response rate (CRR). We excluded peer-reviewed articles, non-original studies, preclinical trials, animal trials, abstract-only publications, and articles in a language other than English. If possible, we tried to contact the authors of studies that were only published as congress abstracts *via* email and asked them to provide their data. The studies selected and all reasons for exclusion are shown in the PRISMA flowchart (**Figure 1**). All included studies were assessed regarding potential conflicts of interest. The present review has been categorized on PROSPERO with code 337174 as an acknowledgment of receipt.

### Data extraction

Data were extracted from the included studies without modifications. The eligibility of studies, inclusion criteria, data extraction, and analysis were independently assessed by two authors (RN and VD). In case of discrepancy, LM decided on inclusion or exclusion. They extracted data on tumor characteristics—size, stage, histological subtype, LVSI status, grading—surgical approach, morbidity, and oncological issues such as recurrences, deaths, RR, and CRR to chemotherapy (CT) regimen. Patients with stage IB to IV were not considered in our population. However, this activity was hindered by different criteria across papers.

### Quality assessment

We assessed the included studies’ quality using the Newcastle-Ottawa scale (NOS) (8). This assessment scale uses three broad factors (selection, comparability, and exposure), with scores ranging from 0 (lowest quality) to 8 (best quality). Two authors (II and MLV) independently rated the studies’ quality. Any disagreement was subsequently resolved by discussion or consultation with CR. We reported the NOS Scale in the **Supplementary Material**.

## Results

Among 168 potentially relevant records, six studies with a total of 124 participants were included in the systematic review and shown in **Table 1** (9–14). Four eligible articles were retrospective studies (9, 10, 13), whereas two articles were prospective trials (9, 10). The PRISMA flow diagram summarizing the selection process is presented in **Figure 1**.

**Table 1:** Characteristics of included studies.

Author, year of publication	Country	Study design	Years	FIGO stage/population	No. of participants	Mean FU (months)
Laurelli et al., 2016 (9)	Italy	Prospective observational monocenter study	2006–2013	IA-G1, G2	21	85.0
Hwang et al., 2017 (10)	Korea	Retrospective observational monocenter study	2011–2015	IA-G2	5	44.4
Chae et al., 2019 (11)	Korea	Retrospective observational monocenter study	2005–2017	IA-G1, G2	71	N/A
Falcone et al., 2020 (12)	Italy	Prospective observational multicenter study	2004–2019	IA-G2	23	35
He et al., 2020 (13)	China	Retrospective observational monocenter study	2005–2019	IA-G2	3	19.5
Andress et al., 2021 (8)	Germany	Retrospective observational monocentric study	2006–2018	IA-G2	1	16

FU, follow-up; FIGO, International Federation of Gynecology and Obstetrics. N/A, Not applicable.

### Outcomes

Andress et al. retrospectively analyzed the course of a patient with early-stage EC who underwent FST shortly after diagnosis. The woman was 35.9 years old, and her body mass index (BMI) was 42.8 kg/m^2^. FST consisted of dydrogesterone 10 mg for 3 months, but she did not respond to the treatment: her disease remained stable after a follow-up (FU) time of 6 months. The woman did not manage to conceive (12). Falcone et al. examined 23 patients with intramucosal, G2 endometrioid EC with a mean FU duration of 35 months (10). The endpoints of their study were CR, RR, and pregnancy and live birth rates. A total of 17 patients (74%) were administered with combined hysteroscopic resectoscope (HR) and levonorgestrel-releasing intra-uterine device (LNG-IUD) (12), megestrol acetate (MA) 160 mg (4), and norethisterone acetate 10 mg (1); four patients (17.4%) were administered with LNG-IUD; one (4.3%) was administered with combined LNG-IUD and oral MA 160 mg; one (4.3%) received oral MA 160 mg (10). After 6, 9, 12, and 13 months from the progestin start date, CR was achieved in different groups of patients with an overall CR rate of 73.9%. The overall RR was 41.1% (7/17). The median duration of CR was 21 months. All patients recurred, but only one underwent definitive surgery. One recurrent case refused surgery and received combined LNG-IUD and MA, obtaining CR in 6 months (10). Among the six patients who did not achieve CR, one had persistence of disease at 6 months and underwent a hysterectomy. The remaining five patients experienced progressive disease and were submitted to definitive surgery (10). At the end of the observation period, 22 patients (95.7%) showed an absence of disease, and one (4.3%) had disease persistence and is alive (10). Regarding pregnancy outcomes, three of 23 women (13% of patients) managed to conceive and had successful pregnancies (10). Hwang et al. evaluated five patients with IA G2 EC treated with medroxyprogesterone acetate (MPA) + LNG-IUD for a mean FU of 44.4 months (13). Three of five women (60%) obtained CR in an average period of 11 months; two patients responded partially; one CR patient tried to conceive through the *in vitro* fertilization (IVF) technique after maintenance therapy for 9 months, but a hysteroscopic endometrial biopsy was performed 14 months after CR detected recurrence (RR = 20%). Hence, she underwent the same treatment with CR after 6 months. After another IVF cycle, her pregnancy resulted in miscarriage (13). He et al. analyzed three patients with stage IA G2 EC treated with MPA + LNG-IUD: they all obtained CR, and one of three recurred (11). Pregnancy outcomes have not been investigated by the authors mentioned above. Chae et al. analyzed 71 patients with stage IA grade 1–2 endometrioid endometrial cancer administered with 500 mg of MAP and LNG-IUD (9). The authors did not distinguish between patients with grades 1 and 2 of the disease, and they noticed that each patient had CR, five of 71 (7.0%) recurred, and 49 women (69.0%) attempted to conceive (9). Among those patients, the overall RR was 36.7% (18 of 49). RR in pregnant women was 18.2% (four of 22) with a mean DFS of 26 months, whereas non-pregnant showed an RR of 51.9% (14 of 27) with 12 months of DFS (9). Laurelli et al. enrolled 21 patients with stage IA grade 1–2 endometrioid endometrial cancer with no grading distinction for a FU period of 85 months on average. Patients were administered HR and diagnostic laparoscopy with ovarian and peritoneal biopsy, which was negative. Then, LNG-IUD was administered for 6 months. Eighteen patients (85.7%) had CR; two of them (9.5%) had persistence of disease, whereas one (4.8%) showed progressive disease at 3 months already; one non-responder refused definitive surgery and underwent the same treatment, obtaining CR. Overall CR rate was 90% and 95% if excluding the G2 patient; 12 CR (63%) had pregnancies. This study revealed CR rates of 95% (19 of 20) with an RR of 10.5% (two of 19) (14). Those data are summarized in **Table 2**.

**Table 2:** Outcomes of IA G2 patients.

Author, year of publication	Treatment	CR	RR	Overall pregnancy rate	Successful pregnancy rate
Hwang et al., 2017 (10)	MPA + LNG-IUD	60.0%	20.0%	20.0%	0.0%
Falcone et al., 2020 (12)	HR + progestin	73.9%	41.1%	13.0%	13.0%
He et al., 2020 (13)	Progestin	100.0%	33%	N/A	N/A
Andress et al., 2021 (8)	Progestin	0.0%	0.0%	0.0%	0.0%

CR, complete response; RR, recurrence rate; HR, hysteroscopic resection; LNG-IUD, levonorgestrel-release intra-uterine device; MPA, medroxyprogesterone acetate; D&C, dilation and curettage. N/A, Not applicable.

## Discussion

Fertility-sparing treatment in EC has been investigated without a defined consensus during the last few years. The difficulty in defining its boundaries may be related to many factors influencing its success. The most important issues are the assessment of the tumor’s clinicopathological biology (histological type, grade, myometrial invasion, and presence of LVSI) and choosing the optimal type, dose, and duration of medical treatment, as well as proper follow-up. Ultimately, the ideal patient presents with minimal disease and minimal risk of distant spread. There is no ideal tool for grading and staging to date. Another fact to consider when proposing an FST is how lymph node status is investigated. In the absence of myometrial infiltration, it is assumed that an imaging method (e.g., CT scan) may be sufficient to exclude suspicion at the lymph node level. The risk of pelvic and/or para-aortic lymph node involvement for grade 1 tumors without myometrial invasion is less than 1% (15). In contrast, there are no data to support a safety profile in patients with EC G2. Similarly, a crucial point in proposing FST is the acquisition of histological specimens. Endometrial biopsy and curettage are the two most reported methods in scientific literature. However, the biopsy may not be representative of the entire tumor specimen. Nevertheless, curettage may hypothetically affect fertility. In addition, the literature reports discrepancies of up to 20% either for curettage or endometrial biopsy, with a higher correlation with the final histological results for curettage (16). They reported an upgrade in the final specimen for G1 EC of 8.7% for curettage and 17.4% for endometrial biopsy. Moreover, it is more likely to completely eradicate the tumor, reduce tumor burden, and facilitate the therapeutic effects of progestins. Chae et al. proved how an augmented frequency of curettages does not influence future pregnancies (9). However, the use of anti-adhesive medications in the curettage technique protects the basal layer of the endometrium. It prevents contact with fibroblasts and hematomas in the healing phase (17–19). Hence, dilation and curettage would be easier to perform mostly if patients were administered progestins for a long period. Although some authors raised awareness of the risk of cytological spread during HR, recent studies demonstrated negative peritoneal cytology in early-stage EC at MRI and TVS performed on surgical specimens, with no impact on prognosis (20, 21). Pregnancy also showed a positive effect on the prognosis of endometrioid endometrial cancer, lowering recurrence rates. Indeed, pregnancy guarantees exposure to endogenous progesterone for a long interval (22). A longer time to recurrence in pregnant women than in non-pregnant women was also reported (22). In the Chae et al. study, the pregnancy rate was notably high, and patients who had pregnancies showed delayed disease recurrence as compared to patients who did not conceive (9). This may suggest the influence of pregnancy-related factors, such as recurrence before pregnancy, endometrial thickness during ovulation, and age at conception (23). For example, in the Laurelli et al. study, one patient had a BMI of 53.5 kg/m^2^, and obesity is a risk factor for endometrial transformation in the context of metabolic syndrome (14). There is evidence that a BMI greater than 25 kg/m^2^ is linked to failure of progestin treatment (22). This suggests that any fertility-sparing protocol should be accompanied by weight loss planning. Preclinical evidence considers whether molecular markers can predict remission and response to therapy in early-stage EC. For example, the expression of progesterone receptor is linked to higher rates of complete remission after MPA treatment (24). Otherwise, progestin therapy showed a response in hormone-negative tumors also, proving the existence of other pathways beyond the interaction with hormonal receptors (25). However, the degree of tumor differentiation is the main predictor of response to hormone therapy. Thigpen et al., since 1999, proved that RR to MPA was 37% compared to 9% in patients with G3 disease (26). Moreover, hormonal treatment can be affected by the expression of the cell adhesion molecule L1-CAM, associated with an invasive pattern of disease, distant metastases, and recurrence (27). Another statement—explaining how tumor grade affects pregnancy—regards plasminogen activator inhibitor type 1, whose levels are lower in stage I EC (28–30). Genetic mutations of PAI-1 lead to infertility (31). Thrombi in EC depend on higher expression of PAI-1, and failed pregnancies may be a direct consequence of this phenomenon. Another area of vulnerability in FST treatment is related to the type of hormone therapy. To date, there are no direct comparison studies between MPA and MA. In a meta-analysis, Koskas reported that MPA shows a higher RR than MA (32). Similarly, the use of LNG-IUD was found to be comparable to oral hormone therapy (33). Moreover, in the literature, there is no univocity even in the dosage and duration of therapies, and the possibility of combining various treatments is not adequately investigated. In 2009, Eftekhar reported a doubled CR following a doubling of the dose of MA (56% *vs.* 28%) (34). However, dual therapy with MPA and LNG-IUD had a higher response compared to single-agent treatment, as demonstrated by an overall complete remission of 87.5% with an average time of 9.8 months by Kim et al. and a complete response of 3/5 patients in the Hwang et al. study (13, 35). The duration of treatment can also affect the CR rate, as reported by Erkanli et al. They observed a CR of 47% in the first 6 months, with an additional 17% between 6 and 9 months and 13% in longer periods (36). Myometrial invasion is another major prognostic factor (15, 26). Although there is no standardization of the best imaging method to evaluate myometrial infiltration, enhanced MRI proved to be the most accurate technique to diagnose myometrial invasion (37), but TVS is also an appropriate method compared to MRI (37). In addition, pelvic MRI assesses endometrium-limited disease, myometrial invasion, and local dissemination, showing more validity compared to ultrasound (US) in detecting lymph node infiltration and metastases (38, 39). In case of persistence or recurrence of disease, the standard of care consists of total abdominal hysterectomy and bilateral salpingo-oophorectomy. The repetition of fertility-sparing techniques—combining HR and LNG-IUD or MPA—may be evaluated in women wishing to maintain their reproductive function, but this scenario is far less investigated. Clinical evidence has noticed from 70% to 85% of complete responses after second-round therapy prolongation up to 12 months. However, those conservative measures must be considered temporary to allow pregnancy and subsequently perform specific counseling to adopt surgery (39–46) finally. A recent systematic review and meta-analysis, performed by Raffone et al., explains that the prognosis of EC could be evaluated according to The Cancer Genome Atlas (TCGA) molecular signature and pathological elements, such as LVSI (44). In particular, LVSI, age, and adjuvant therapy have a critical prognostic value, increasing death and disease progression of EC up to two times (47). Molecular prognostic factors may be applied also to predict the efficacy of FST of EC in clinical practice. Raffone et al. found that deep myometrial invasion (DMI) is not independent of TGCA in determining OS in EC, but it independently influences RR (48). Our opinion is that the study’s strength lies in its systematic nature and rigor in searching and extracting all the literature data about EC IA G2 patients for the first time. Similarly, this represents the main limitation of our paper, which aims to summarize data from extremely heterogeneous approaches and populations of patients that, however, reflect well the current clinical practice. In addition, this review is a partial view of the problem of fertility preservation, mainly focused on oncological outcomes. Novel pieces of evidence confirm that patients with reproductive potential with stage IA G2 EC are candidates for FST: in particular, progestins are a valid option for endometrioid histotypes without myometrial invasion (44, 49, 50). Casarin et al. underline the potential role of glandular cells (GCs) in preoperative cervical smear for diagnosis and management of early-stage EC (51). This may be useful in clinical practice to predict local recurrence in women administered with FST. Moreover, Tanos et al. identified molecular signatures as prognostic elements in FST: PTEN is a favorable factor in FST administration, and K-RAS is associated with recurrence, regardless that PIK3CA, HER2, and P53 have a poor prognostic value (52). After FST techniques, both open and closed vitrification methods for blastocyst embryo transfer would increase the pregnancy rate (53). Although myometrial infiltration has often been considered an exclusion criterion for conservative techniques, recent findings suggest that women with minimally infiltrating G1 EC could be administered with FST (54). FU may include endometrial biopsies every 3 months for 1 year and every 6 months for the following 4 years (54). Moreover, Casadio et al. treated three patients affected by G1 endometrioid EC with HR and hormone therapy (55). The 5-year FU was negative for neoplasia, and two of three patients achieved pregnancy (55). It seems clear that greater standardization in the selection of patients is necessary, and a risk classification even within a pattern of patients—with EC IA G2—is already considered at extreme limits of acceptability in FST. The higher the patient’s inherent risk, the more attention should be paid by the physician to the contextualization of the proposed clinical pathway. Therefore, it would be desirable to design clinical trials that prospectively minimize the bias related to tumor characteristics and not to the proposed FST. In conclusion, fertility-sparing management is not the current standard of care for young women with EC and can be employed for patients with early-stage G1 EC motivated to maintain reproductive function. Otherwise, this management is not the gold standard in EC, and it would be appropriate to plan specific counseling for patients undergoing this experimental approach for fertility preservation, although the ideal fertility-sparing treatment of EC is not yet defined. Further evidence is needed to investigate the actual benefit.

## Data availability statement

The original contributions presented in the study are included in the article/**Supplementary Material**. Further inquiries can be directed to the corresponding author.

## Author contributions

Supervision: LM, FP. Conceptualization: CR, GV, SR. Methodology: MT, NC. Writing, Original Draft: II, RN, DV. Writing, Review and Editing: II, RC, MS, PF, MV. All authors contributed to the article and approved the submitted version.

## Conflict of interest

The authors declare that the research was conducted in the absence of any commercial or financial relationships that could be construed as a potential conflict of interest.

## Publisher’s note

All claims expressed in this article are solely those of the authors and do not necessarily represent those of their affiliated organizations, or those of the publisher, the editors and the reviewers. Any product that may be evaluated in this article, or claim that may be made by its manufacturer, is not guaranteed or endorsed by the publisher.
