# Supplementary material for: Oncological outcomes in fertility-sparing treatment in stage IA-G2 endometrial cancer
Source: Front Oncol. 2022 Sep 16;12:965029. doi: 10.3389/fonc.2022.965029 (PMC9524219; doi:10.3389/fonc.2022.965029)
Supplement: Supplementary file 1 [file Table_1.docx]

**Supplementary Table 1.** Quality scores of included studies, assessed by the Newcastle-Ottawa Scale.

|  | **Selection** | | | | **Comparability** | | **Exposure** | | |  |
| --- | --- | --- | --- | --- | --- | --- | --- | --- | --- | --- |
| **Author, year** | **Case definition** | **Representative-ness of the cases** | **Selection of Controls** | **Definition of Controls** | **Control for the most important factor** | **Control for any additional factors** | **Ascertainment of exposure** | **Same method for cases and controls** | **Non-Response Rate** | **Overall quality** |
| Laurelli et al., 2016^13^ | 1 | 0 | 0 | 0 | 0 | 0 | 1 | 0 | 1 | 3/8 |
| Hwang et al., 2017^10^ | 1 | 1 | 0 | 0 | 0 | 0 | 1 | 0 | 1 | 4/8 |
| Chae et al. 2019^12^ | 1 | 0 | 0 | 0 | 0 | 0 | 1 | 0 | 1 | 3/8 |
| Falcone et al., 2020^9^ | 1 | 1 | 0 | 0 | 0 | 0 | 1 | 0 | 1 | 4/8 |
| He et al., 2020^11^ | 1 | 1 | 0 | 0 | 0 | 0 | 1 | 0 | 1 | 4/8 |
| Andress et al., 2021^8^ | 1 | 1 | 0 | 0 | 0 | 0 | 1 | 0 | 1 | 4/8 |
